# Temperature-Controlled Transportation Preserves Hot Fresh Pork Quality: The Balance Between Color Stability and Shelf-Life

**DOI:** 10.3390/foods15030444

**Published:** 2026-01-26

**Authors:** Jiaxin Wang, Ge Song, Shaolin Deng, Xiaoming Wang, Dongling Li, Xiaozhi Wang, Guanghong Zhou, Chong Wang

**Affiliations:** 1 State Key Laboratory of Meat Quality Control and Cultured Meat Development, Key Laboratory of Meat Products Processing, Ministry of Agriculture, Jiangsu Collaborative Innovation Center of Meat Production and Processing, Quality and Safety Control, College of Food Science and Technology, Nanjing Agricultural University, Nanjing 210095, China; wangjiaxin@stu.njau.edu.cn (J.W.);; 2 Wens Foodstuff Group Co., Ltd., Yunfu 527400, China

**Keywords:** hot fresh pork, temperature-controlled transportation, quality, color, bacterial community succession

## Abstract

Hot fresh pork is highly preferred by Chinese consumers for its desirable flavor and color. However, its quality deteriorates rapidly during ambient-temperature transportation, leading to unappealing meat color and shortened shelf life. This study investigated the effects of different transportation temperature setpoints (5 °C, 10 °C, 15 °C Setpoint groups, and ambient temperature) on pork carcass quality. Transportation at the lower setpoints (5 °C, 10 °C) reduced carcass center temperature, attenuated pH decline, minimized cooking and drip losses, suppressed microbial proliferation, and curtailed TVB-N accumulation (*p* < 0.05). These conditions also shortened the duration of high temperatures in vehicles, decelerated glycogenolysis, and moderated energy metabolism, collectively preserving meat quality. Regarding color, 5 °C Setpoint group inhibited myoglobin oxidation, yielding lower oxygenated myoglobin content and reduced a* values compared with 10 °C Setpoint group over 150 km (*p* < 0.05). High-throughput sequencing revealed that temperature setpoint transportation significantly influenced bacterial community succession, with highly similar profiles between the 5 °C and 10 °C Setpoint groups, yet clear divergence from the ambient control. Therefore, transportation at 10 °C Setpoint represents a balanced approach to preserving color, delaying spoilage, and extending shelf life.

## 1. Introduction

Pork plays a critical role in China’s agricultural economy as the world’s largest producer and consumer of pork [1]. Unlike Western countries, where chilled pork accounts for more than 90% of consumption, China’s market is dominated by hot fresh pork (sold immediately without sufficient cooling or subjected only to very brief pre-cooling), which comprises 60% of total pork consumption, while chilled pork represents less than 30% [2]. Hot fresh pork is highly preferred by Chinese consumers due to its flavorful texture and reddish color. However, current transportation methods present significant challenges to maintain these quality attributes of hot fresh pork.

In China, hot fresh pork is typically transported in non-refrigerated, open vehicles without temperature control or standardized management. This uncontrolled ambient transportation, especially during summer, accelerates microbial growth and quality deterioration (evidenced by increased drip loss, color fading, reduced consumer acceptance). Consequently, hot fresh meat distribution remains geographically constrained, largely confined to local markets near slaughterhouses, which severely curtails its broader market potential [3]. Therefore, developing effective long-distance transportation protocols could yield substantial economic and ecological benefits by reducing redundant regional slaughterhouse construction, expanding market coverage, and minimizing environmental pollution (wastewater, exhaust emissions, solid waste), which is in line with low-carbon agriculture objectives.

Post-slaughter biochemical processes, particularly muscle energy metabolism, play a pivotal role in determining ultimate meat quality [4]. Glycolysis emerges as a critical post-slaughter physiological process [5], with glycolytic enzymes serving as key regulators [6]. Studies have shown that muscle glycogen content and glycolytic enzyme activities significantly influence various meat quality including tenderness, color stability, water-holding capacity through their roles in the glycolytic pathway [7]. Notably, glycogen phosphorylase and pyruvate kinase serve as key regulators of post-slaughter glycolysis, with their activities being closely associated with the development of pale, soft, exudative (PSE) meat [4]. However, the extent to which transportation temperature regulates glycolysis and its subsequent impact on the quality of hot fresh pork remain incompletely understood.

Microbiological safety is another crucial factor during transportation. Microbial growth is a primary cause of quality deterioration [8,9], and inappropriate transportation conditions can thus lead to a decline in physicochemical quality, thereby ultimately threatening consumer health [10]. While the cold chain is established as the foremost preservation method, effectively curbing microbial and enzymatic spoilage to prolong shelf life [11], it introduces a dilemma. Excessively low temperatures may inadvertently affect color stability—a key consumer acceptability parameter—by inhibiting myoglobin oxygenation or metmyoglobin reductase activity, leading to undesirable darkening [12,13]. Thus, identifying an optimal transportation temperature that ensures both microbial safety and visual appeal is essential. Importantly, the current studies are heavily centered on chilled and frozen meat products. There is a pronounced lack of research dedicated to the quality management of hot fresh pork during transportation. Given its high initial temperature and consequently greater vulnerability to microbial spoilage and biochemical changes, investigating tailored transport protocols for hot fresh meat represents a significant and under-explored area of research.

Based on the above research background, we hypothesize that moderate low-temperature transportation would balance the delay of post-mortem glycolysis, inhibition of microbial proliferation, and preservation of desirable redness, thereby enhancing overall meat quality and shelf-life. This study systematically evaluates the effects of different transportation temperature setpoints (5 °C Setpoint, 10 °C Setpoint, 15 °C Setpoint, and ambient) on hot fresh pork quality parameters including pH, temperature profile, drip loss, cooking loss, meat color, TVB-N values and surface bacterial counts. Through integrated analysis of glycolytic metabolism and high-throughput sequencing technology, we elucidate the underlying mechanisms governing quality changes during transportation. Our results provide a scientific basis for designing long-distance transportation strategies that simultaneously preserve sensory appeal and ensure microbiological safety, thereby facilitating the market expansion of hot fresh pork.

## 2. Materials and Methods

### 2.1. Sample Transportation and Collection

Forty castrated male pigs (Duroc × Landrace × Yorkshire, 6 months, 85 ± 10 kg) were selected randomly and slaughtered in a commercial slaughterhouse (Guangdong, China). After slaughter, carcasses were placed in a 4 °C chilling room for brief pre-cooling (20 min), and then immediately transferred and suspended in the transport vehicle. Following identification based on weight range, they were randomly assigned to one of four transportation temperature conditions (*n* = 10 per group) using a computer-generated random number sequence. The groups were designated according to the setpoint of the cooling system in the transportation compartment: the 5 °C Setpoint group, 10 °C Setpoint group, 15 °C Setpoint group, and ambient temperature control group (Con). The transportation was conducted from 2:00 to 6:00 AM during summer (July 2025) to simulate common commercial practices. Carcasses were transported for 150 km maintaining an approximate speed of 60 km/h. The actual temperature and relative humidity within the transport compartment were monitored continuously throughout the whole process. Samples were collected from both sides of the *Musculus longissimus thoracis et lumborum* (LTL) at transportation distances (time) of 0 km, 50 km, 100 km and 150 km (corresponding to approximately 0, 50, 120, and 180 min, respectively, as the total time included standardized 15-min sampling stops at each distance point) for subsequent analysis.

### 2.2. Vehicle Temperature and Humidity Monitoring

Prior to carcass loading, temperature and humidity loggers (Testo 174T, Testo SE & Co. KGaA, Lenzkirch, Germany) were secured at distinct locations beneath the vehicle ceiling. These loggers continuously monitored compartment conditions throughout transportation.

### 2.3. Carcass Temperature Measurement

A penetration thermometer (Testo 106, Testo SE & Co. KGaA, Lenzkirch, Germany) was inserted perpendicularly into the *semimembranosus* of each pig carcass to a depth of 3–5 cm for core temperature measurement. Three measurements were taken at several different locations within each carcass.

### 2.4. pH Measurement

pH was measured using a portable pH meter (Testo 205, Testo SE & Co. KGaA, Lenzkirch, Germany) following the method described by Yan et al. [14]. The meter was calibrated before use by standard buffers (pH 4.00 and 7.00). For each carcass, triplicate readings were collected from at least three different locations within the longissimus thoracis, with the pH probe inserted approximately 2 cm into the muscle at each point.

### 2.5. Microbial Colony Counts on the Surface of Pig Carcasses

Surface bacterial counts were determined according to Wu et al. [15]. Briefly, at each sampling distance, surface microorganisms were collected from pig carcasses by swabbing the breech surface (100 cm^2^) with a sterile swab containing a peptone solution (0.1%). The collected samples were serially diluted, inoculated onto Plate Count Agar (PCA, Haibo Biotechnology Co., Ltd., Changsha, China) plates, and incubated at 37 °C for 48 h. Colony counts were then enumerated and expressed as log_10_ colony-forming units per square centimeter (CFU/cm^2^).

### 2.6. Determination of Total Volatile Basic Nitrogen (TVB-N)

TVB-N content was determined according to the Chinese National Standard GB 5009.228-2016 [16]. Specifically, each sample of 10 g was homogenized with 75 mL distilled water for 30 min, followed by addition of 1 g of magnesium oxide (MgO). The TVB-N value was quantified using an automatic Kjeldahl Nitrogen determination apparatus (K1160, Hanon Instruments, Jinan, China) and calculated with the following equation:

(1) $$\text{TVB-N}\left(\frac{mg}{100\;g}\right)=\frac{\left(V_{1}-V_{2}\right)\times c\times 14}{m}\times 100$$

where V_1_ and V_2_ represent the volume (mL) of the hydrochloric acid consumed by the sample and blank. c represents the exact concentration (mol/L) of hydrochloric acid. m represents sample weight (g).

### 2.7. Determination of Cooking Loss

Individual samples (50.00 ± 0.05 g) were sealed in cooking bags and heated in a water bath maintained at 72 °C until the core temperature of the samples reached 70 °C. The cooked samples were immediately cooled under running water to room temperature, gently blotted dry with filter paper, and reweighed. The percentage of cooking loss was calculated according to the following equation:

(2) $$Cooking\;loss\left(\%\right)=\frac{\left(m_{1}-m_{2}\right)\times 100}{m_{1}}$$

where m_1_ and m_2_ represent the weight (g) of the sample before and after cooking, respectively.

### 2.8. Determination of Drip Loss

Drip loss was determined using meat samples cut into cuboids (2 cm × 3 cm × 5 cm). Each sample was vacuum-sealed in a plastic bag, hung at 4 °C for 24 h, and then reweighed. The drip loss percentage was calculated according to the following equation:

(3) $$Drip\;loss\left(\%\right)=\frac{\left(m_{3}-m_{4}\right)\times 100}{m_{3}}$$

where m_3_ and m_4_ represent the weight (g) of the sample before and after hanging, respectively.

### 2.9. Color Measurement

Meat color was determined using a CR-400 colorimeter (Konica Minolta Sensing Corporation, Tokyo, Japan) according to the method described by Okpala et al. [17]. The instrument was calibrated with a certified white reference tile prior to analysis. The lightness and redness of the samples were expressed as CIE L* and a*, respectively. Measurements were taken at three locations on the freshly cut surface of each longissimus thoracis muscle sample after a standardized 20-min blooming period.

### 2.10. Quantification of Oxymyoglobin Content

Oxymyoglobin (OxyMb) content was evaluated based on a protocol described by Li et al. [18] with minor modifications. Briefly, 2 g of meat was homogenized with 25 mL of ice-cold phosphate buffer (0.04 M, pH 6.8) for 1 min at 8000 rpm using a homogenizer (IKA T25 digital ULTRA-TURRAX, Staufen, Germany). The resulting homogenate was incubated at 4 °C for 1 h, followed by centrifugation at 10,000× *g* for 30 min (4 °C), and filtered. Subsequently, 300 μL of the filtrate was transferred to a microplate well. Absorbance was measured at 525, 545, 565, and 572 nm using a full-wavelength microplate reader (Spark, Tecan Group Ltd., Männedorf, Switzerland). OxyMb content was calculated using the following equation:

(4) $$OxyMb\left(\%\right)=\left(0.882R_{1}-1.267R_{2}+0.809R_{3}-0.361\right)\times 100$$

where R_1_ = A_572_/A_525_, R_2_ = A_565_/A_525_, R_3_ = A_545_/A_525_.

### 2.11. Quantification of Glycogen, Lactic Acid, ATP Content

Glycogen, L-lactic acid, and ATP contents were quantified using commercial kits (Solarbio, Beijing, China) according to manufacturers’ protocols. Specifically, glycogen content was measured with kit BC0345, L-lactic acid with kit BC2235, and ATP with kit BC0305. Sample preparation and analyses were performed following the respective kit specifications.

### 2.12. Quantification of Glycogen Phosphorylase and Pyruvate Kinase Activities

The activities of glycogen phosphorylase (GP) and pyruvate kinase (PK) were quantified using commercial enzyme assay kits (Glycogen Phosphorylase Kit, BC3345; Pyruvate Kinase Kit, BC0545; Solarbio, Beijing, China) according to the manufacturer’s protocol.

### 2.13. Bacterial Community Analysis

Total genomic DNA from the above-mentioned swabbed samples was extracted using the E.Z.N.A.^®^ Tissue DNA Kit (Omega Bio-tek, Norcross, GA, USA) according to manufacturer’s protocols. DNA concentration and purity were assessed by 2% agarose gel electrophoresis and spectrophotometry (NanoDrop 2000, Thermo Fisher Scientific, Waltham, MA, USA). The V3-V4 hypervariable region of the bacterial 16S rRNA gene was amplified via PCR with the universal primers 341F (5′-CCTAYGGGRBGCASCAG-3′) and 806R (5′-GGACTACHVGGGTWTCTAAT-3′). All amplicons were sequenced on the MGI-G99 platform by Biozeron Technology Co., Ltd. (Shanghai, China).

### 2.14. Statistical Analysis

Statistical analyses of carcass temperature, pH value, TVB-N, water-holding capacity, color and oxymyoglobin content, ATP, glycogen and lactic acid content, glycolytic enzyme activity were performed with IBM SPSS Statistics 26.0 (IBM Corporation, Armonk, NY, USA). Significant differences among treatments were analyzed by one-way analysis of variance (ANOVA) and Duncan’s multiple comparisons with a significance threshold set at *p* < 0.05. Data are reported as means ± standard deviation (SD). Graphical representations were generated using GraphPad Prism 8.0 (GraphPad Software, Boston, MA, USA).

For microbial analysis, the α-diversity (within-sample diversity) was estimated using the Shannon index based on the abundance of reads at the ASV level. The β-diversity analysis between groups was performed and visualized by the principal coordinate analysis (PCoA) using Bray–Curtis distance matrices based on the ASV level (vegan package, version 2.5–7, R software, version 4.0.2, Jari Oksanen, Helsinki, Finland).

A linear discriminant analysis effect size (LEfSe) analysis was performed to identify bacterial biomarkers in hot fresh meat transported at different temperatures over a distance of 150 km. The Kruskal–Wallis sum-rank test was performed for statistical analysis and a LDA analysis was then applied to determine the effect size of each distinctively abundant taxa (microbiomeMarker package, version 0.99.0, R software, Yang Cao, Tianjin, China).

## 3. Results and Discussion

### 3.1. Temperature and Humidity Profiles of Vehicle

**Figure 1:**
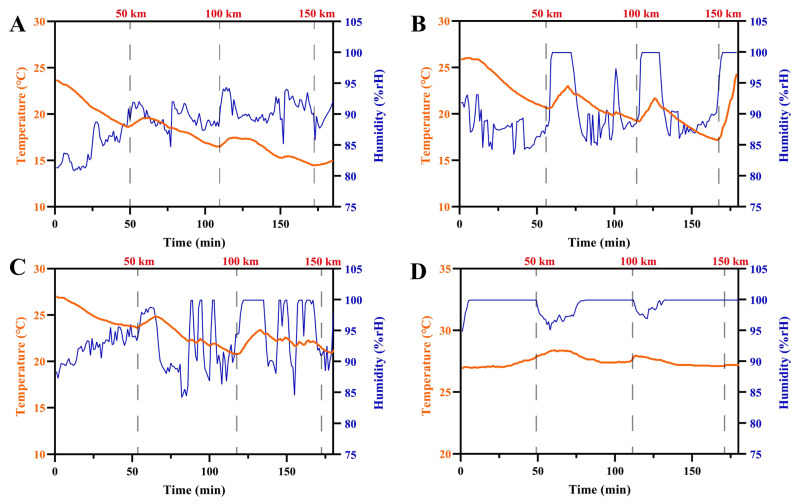
Changes in temperature and humidity in the transportation vehicle compartment under different temperature-controlled conditions. (**A**–**D**) Comparisons of temperature and humidity profiles during transportation under controlled conditions of 5 °C Setpoint Group (**A**), 10 °C Setpoint Group (**B**), 15 °C Setpoint Group (**C**) and ambient temperature (**D**).

Temperature and humidity are critical factors for maintaining pork quality and safety, especially in inhibiting bacterial proliferation [19,20,21]. During the 150 km transportation (approximately 3 h), vehicle temperature ranges were 14.5–23.7 °C (5 °C Setpoint group), 17.1–25.9 °C (10 °C Setpoint group), 20.7–27.0 °C (15 °C Setpoint group), and 26.9–28.4 °C (control) (Figure 1A–D). Corresponding relative humidity (RH) values were 80.90–97.1%, 84.3–99.9%, 84.15–99.9%, and 95.9–99.9%, respectively. Notably, the control group exhibited the highest vehicle temperature and RH, likely attributable to summer ambient conditions. Throughout the transportation, a consistent deviation was observed between the set temperature and the actual cabin temperature across all treatment groups. This deviation primarily resulted from substantial metabolic heat released by the pork carcasses (initial carcass temperature ~39–40 °C) coupled with inherent limitations in refrigeration system efficiency and routine heat exchange during transportation.

### 3.2. Carcass Temperature and pH Evolution

**Figure 2:**
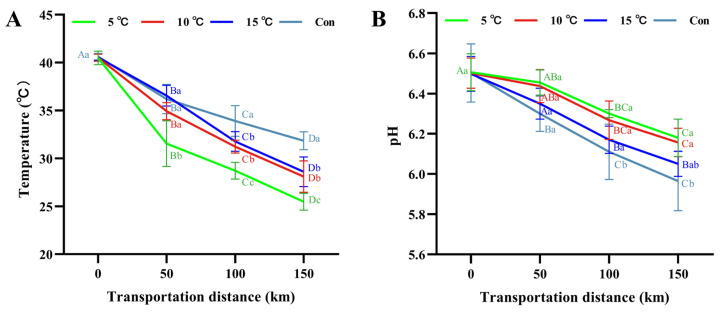
Changes in pork carcass temperature (**A**) and pH (**B**) under different transportation temperature setpoint conditions. Different uppercase letters indicate significant differences among transportation distances at the same temperature (*p* < 0.05). Different lowercase letters indicate significant differences among transportation conditions at the same distance (*p* < 0.05).

Initial carcass temperature was 40.5 °C due to insufficient pre-cooling of hot fresh pork carcass [3], decreasing with transport duration (*p* < 0.05) (Figure 2A). Carcass temperature trends mirrored compartment conditions. The 5 °C Setpoint group achieved the lowest final core temperature 25.5 °C vs. 28.1 °C (10 °C Setpoint group), 28.6 °C (15 °C Setpoint group), and 31.8 °C (control), with the control being higher (*p* < 0.05). Given the established correlation between prolonged high temperature of carcass in early post-slaughter phases, rapid pH decline and increased PSE meat incidence [22], our results indicated that ambient transportation elevated the risk for developing PSE-like characteristics.

Post-slaughter pH evolution influences pork texture, water-holding capacity, and spoilage susceptibility [23]. As shown in Figure 2B, the initial pH was 6.50, and a decreasing trend was observed across all groups as transportation distance increased. After 150 km of transportation, pH values decreased to 6.18, 6.16, and 6.05 for the 5 °C Setpoint, 10 °C Setpoint, and 15 °C Setpoint groups, respectively. In contrast, the control group exhibited a lower pH of 5.97 (*p* < 0.05) compared with the 5 °C Setpoint and 10 °C Setpoint groups. This accelerated pH decline in the control group may be attributed to higher early post-slaughter carcass temperatures, which likely accelerated glycolytic rates. It has been reported that rapid cooling can effectively suppress anaerobic muscle metabolism, especially glycolysis in pork muscle [24]. Anaerobic fermentation produces lactic acid, and the breakdown of ATP releases phosphate ions, both of which cause the pH decline [25]. Therefore, transportation at 5 °C Setpoint and 10 °C Setpoint effectively delayed pH decline, contributing to better maintenance of hot fresh pork quality.

### 3.3. Surface Microbial Colony and TVB-N

**Figure 3:**
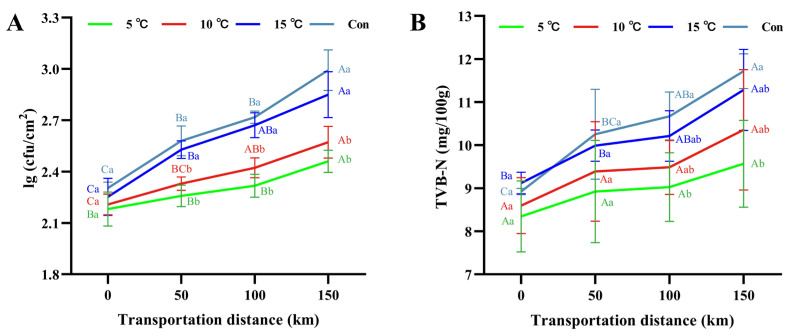
Changes in surface microbial colony (**A**) and TVB-N (**B**) of hot fresh pork under different transportation temperature setpoint conditions. Different uppercase letters indicate significant differences among transportation distances at the same temperature (*p* < 0.05). Different lowercase letters indicate significant differences among transportation conditions at the same distance (*p* < 0.05).

Microorganisms utilized carbohydrates, proteins, and fats in raw meat to produce metabolites such as organic acids, amines, ammonia, aldehydes, and ketones that compromise meat quality [26]. As shown in Figure 3A, microbial colony counts on the carcass surface increased with transportation distance. Initial counts were consistent across all groups, averaging 2.24 ± 0.05 lg CFU/cm^2^. After 150 km transportation, counts reached 2.46 ± 0.06, 2.57 ± 0.09, 2.85 ± 0.13, and 2.99 ± 0.12 lg CFU/cm^2^ for the 5 °C Setpoint, 10 °C Setpoint, 15 °C Setpoint, and control groups, respectively. Due to the relatively short transportation duration, the final counts in all groups of this study remained below the satisfactory limit of 4.0 lg CFU/cm^2^ (Commission Regulation (EC) No 2073/2005 [27]). Nevertheless, counts in the 15 °C Setpoint and control groups were higher than those in the 5 °C and 10 °C Setpoint groups (*p* < 0.05). This pattern can be attributed to the combined effect of elevated temperature and high humidity in the 15 °C Setpoint and control groups, which provided favorable conditions for microbial growth and proliferation. This finding aligned with previous studies [19,21], suggesting that low-temperature transportation effectively suppresses microbial growth, while also highlighting the role of environmental humidity in modulating microbial dynamics during transportation.

TVB-N is a critical indicator widely employed to evaluate meat freshness [28]. As shown in Figure 3B, TVB-N values increased over transportation across all groups. This rise can be attributed to extensive microbial growth and subsequent protein degradation, which leads to the accumulation of alkaline nitrogenous compounds, consequently generating TVB-N and reducing pork freshness. Previous studies indicate that lower temperatures can impede bacterial proliferation and delay protein degradation [29,30]. Consistent with these reports, the TVB-N values of the 5 °C and 10 °C Setpoint groups remained relatively stable throughout transportation, whereas a marked increase was observed in the 15 °C Setpoint and control groups. At 150 km, the TVB-N value in the control group reached 11.72 mg/100 g, which was higher (*p* < 0.05) than that in the 5 °C Setpoint group (9.57 mg/100 g). It is noteworthy that all measured values were below the standard limit of 15 mg/100 g (Chinese Standard GB 2707-2016, 2016 [31]). These results demonstrated a clear correlation between transportation temperature and the elevation in TVB-N values, underscoring the role of low-temperature transportation in delaying protein degradation and extending meat shelf life.

### 3.4. Water-Holding Capacity

**Figure 4:**
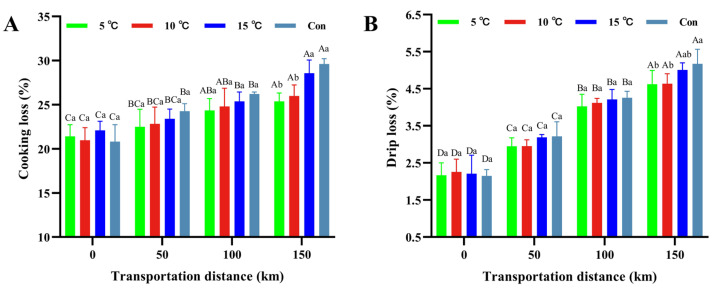
Changes in cooking loss (**A**) and drip loss (**B**) of hot fresh pork under different transportation temperature setpoint conditions. Different uppercase letters indicate significant differences among transportation distances at the same temperature (*p* < 0.05). Different lowercase letters indicate significant differences among transportation conditions at the same distance (*p* < 0.05).

Water-holding capacity represented a critical meat quality parameter [32,33]. Cooking loss and drip loss substantially influence consumer acceptability, sensory traits such as taste and flavor, product yield, and are closely associated with muscle microstructure and texture [34,35]. During 150 km transportation, both cooking loss and drip loss progressively increased across all experimental groups (Figure 4A,B). At the end of transportation, the cooking losses reached 28.57% (15 °C group) and 29.62% (control group), which were higher (*p* < 0.05) than those observed in the 5 °C Setpoint (25.38%) and 10 °C Setpoint (26.00%) groups (Figure 4A). Similarly, the drip loss was highest in the control group (5.17 ± 0.4%), compared with the 15 °C Setpoint group (*p* > 0.05), but was higher (*p* < 0.05) than 5 °C and 10 °C Setpoint groups (Figure 4B). These increased losses were attributed to rapid post-slaughter pH decline impairing pork protein functionality, combined with high-temperature promoting microbial proliferation and proteolytic degradation, ultimately diminishing WHC [36]. Crucially, the 5 °C Setpoint group and 10 °C Setpoint group transportation groups exhibited significantly attenuated increases in both cooking and drip losses, indicating that a low-temperature transportation environment effectively helped maintain WHC of hot fresh pork, which was consistent with previous study [2].

### 3.5. Color and Oxymyoglobin Content

**Figure 5:**
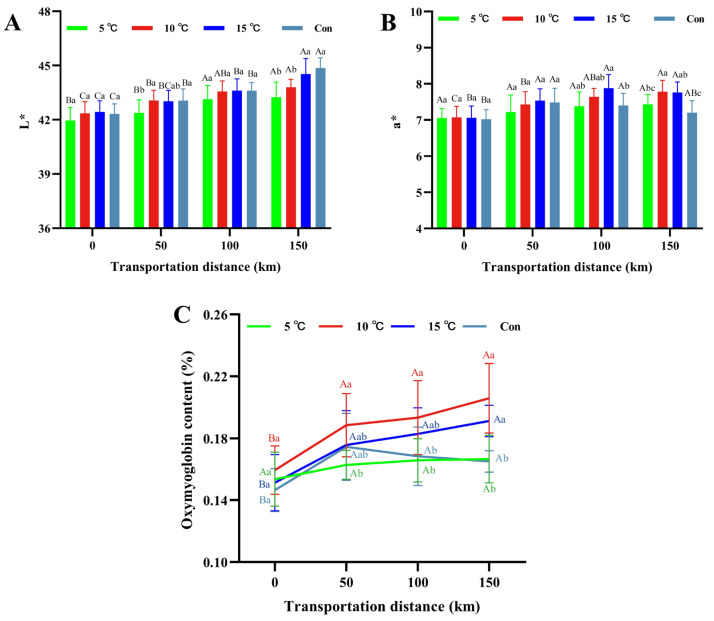
Changes in L* value (**A**), a* value (**B**) and oxygenated myoglobin (**C**) of hot fresh pork under different transport temperature setpoint conditions. Different uppercase letters indicate significant differences among transportation distances at the same temperature (*p* < 0.05). Different lowercase letters indicate significant differences among transportation conditions at the same distance (*p* < 0.05).

The color of fresh pork, a critical determinant of consumer preference, is primarily governed by the content and redox state of myoglobin [37,38]. As shown in Figure 5A, the L* values of pork increased during transportation across all temperature groups, which may be caused by enhanced light reflectance resulting from moisture exudation [39]. The 15 °C Setpoint group and control groups exhibited higher L* values than 5 °C and 10 °C Setpoint groups (*p* < 0.05), indicating greater moisture loss under high temperatures. This accelerated moisture loss is consistent with the observed decline in water-holding capacity (Section 3.4) and is attributed to enhanced physiological and biochemical reactions accelerating myofibrillar protein degradation [39].

The a* value is a critical visual attribute influencing consumers’ purchasing decisions [37]. During 150 km transportation, the a* value in the control group exhibited an initial increase followed by a decrease, a pattern that is consistent with the typical post-mortem color trajectory observed in pork, as reported by Liu et al. [40] in both RFN and PSE meat during aging. The ultimate a* value in the control group (7.20) was significantly lower (*p* < 0.05) than that in the 10 °C Setpoint (7.78) and 15 °C Setpoint (7.76) groups. This pattern may be explained by the oxidation promoted by the high-temperature environment. Elevated temperatures accelerated post-slaughter metabolism, leading to a rapid decrease in pH. The resultant acidic environments promoted myoglobin denaturation, diminishing its oxygen-binding capacity and consequently reducing a* values [41]. No significant difference in a* value was observed between 10 °C Setpoint and 15 °C Setpoint groups (*p* > 0.05), suggesting that this temperature range might be optimal for maintaining color stability during transportation. Conversely, the 5 °C Setpoint group maintained relatively stable a* value during transportation but ended with a lower a* value (7.43) (*p* < 0.05) than the 10 °C Setpoint group. This intriguing result was explained by a dual effect of low temperature: while it slowed metabolic and oxidative processes, it also inhibited myoglobin oxygenation, which is a process essential for the formation of bright-red oxymyoglobin [12]. The delayed color development in our 5 °C Setpoint group aligned with the kinetics observed by Liu et al. [40], who reported the a* value peak at day 1 under 4 °C storage. Given that our transportation study spanned only 3 h—a much shorter timeframe than the intervals in Liu’s aging study—it was consistent that the a* value in the 5 °C Setpoint group had not yet reached its peak. This supported the view that low temperature delayed the manifestation of meat color attributes, and our findings under dynamic transportation conditions further specified that this delay occurred within the critical early post-mortem hours.

Changes in oxygenated myoglobin (Oxy Mb) content during transportation are shown in Figure 5C. At 150 km, Oxy Mb levels in the control and 5 °C Setpoint groups were lower (*p* < 0.05) than those in the 10 °C and 15 °C groups. The control group exhibited an initial increase followed by a decline in Oxy Mb relative content, consistent with the observed a* value trends. This pattern may result from the higher ambient temperature and humidity promoting myoglobin oxidation or denaturation [42]. Conversely, Oxy Mb content in 5 °C Setpoint group remained stable throughout transportation. These results demonstrate that low temperature inhibit myoglobin conversion rate, whereas high temperature accelerated its oxidation or denaturation.

### 3.6. ATP, Glycogen and Lactic Acid Content

**Figure 6:**
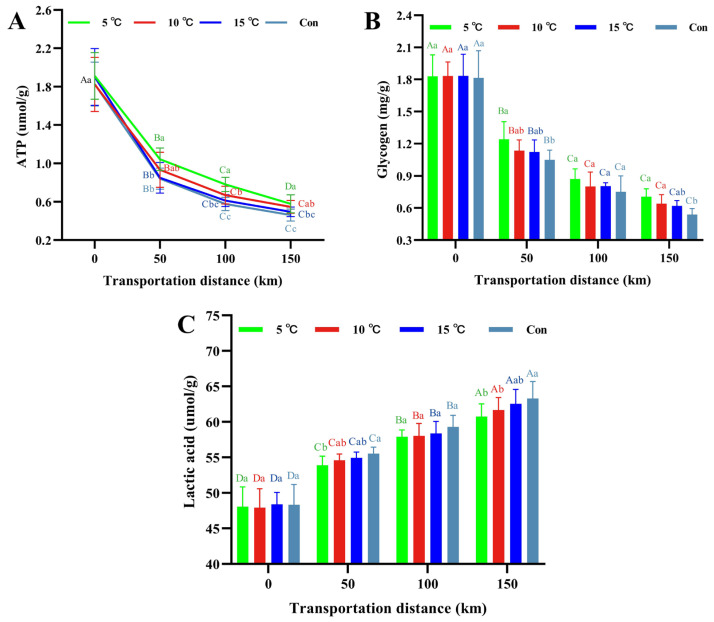
Changes in ATP (**A**), glycogen (**B**) and lactic acid content (**C**) of hot fresh pork under different transport temperature setpoint conditions. Different uppercase letters indicate significant differences among transportation distances at the same temperature (*p* < 0.05). Different lowercase letters indicate significant differences among transportation conditions at the same distance (*p* < 0.05).

ATP levels significantly influence meat quality. As shown in Figure 6A, ATP content decreased in all groups during transportation (*p* < 0.05), with significant differences among groups (*p* < 0.05). This decline results from the rapid shift from aerobic to anaerobic respiration in the early post-slaughter period, during which ATP is consumed to maintain cellular energy. The control group exhibited the most rapid ATP depletion, with its content decreasing to 0.46 µmol/g at the end of transportation, which was lower (*p* < 0.05) than the 10 °C Setpoint group (0.55) and the 5 °C Setpoint group (0.58), which may be attributed to enhanced enzyme activity and accelerated energy metabolism at high temperature, promoting ATP degradation and contributing to rapid pH decline [24].

As the primary energy storage substrate, muscle glycogen is broken down into glucose to fuel glycolysis, which is a pathway that generates ATP under anaerobic conditions [43]. As shown in Figure 6B, muscle glycogen decreased (*p* < 0.05) across all groups during transportation, with temperature-dependent differences (*p* < 0.05). At the endpoint of transportation, glycogen content in the 5 °C Setpoint group (0.70 mg/g) and the 10 °C Setpoint group (0.64 mg/g) was higher (*p* < 0.05) than that in the control group (0.54 mg/g), indicating a slower glycogen consumption rate in the low-temperature transportation groups, which resulted in higher residual glycogen levels in meat [44].

As the end-product of anaerobic glycogen metabolism, lactic acid accumulation directly affects muscle pH and tenderness in post-slaughter carcasses [43]. Lactic acid content increased with transport distance (Figure 6C), reflecting progressive anaerobic respiration. At 150 km, lactic acid content was similar between 5 °C Setpoint (60.73 µmol/g) and 10 °C Setpoint (61.68 µmol/g) groups (*p* > 0.05) but lower (*p* < 0.05) than that in the control group (63.31 µmol/g). The results showed that low-temperature transportation reduced lactic acid accumulation, which in turn attenuated the decline in muscle pH [45], which is consistent with observed pH trends. The significantly greater glycogen depletion and lactic acid accumulation in the control group, compared to 5 °C and 10 °C Setpoint groups indicate accelerated glycolysis. Collectively, these findings demonstrate that low-temperature transportation suppresses the rate of energy metabolism, delays the degradation of ATP and glycogen, reduces lactic acid accumulation, and ultimately contributes to improved quality of hot fresh meat.

### 3.7. Glycolytic Enzyme Activity

**Figure 7:**
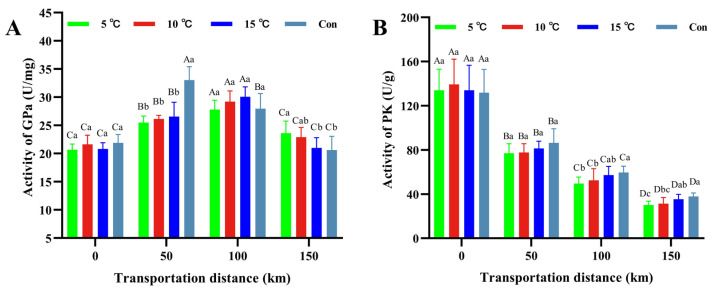
Changes in Glycogen phosphorylase-a enzyme activity (**A**) and Pyruvate kinase activity (**B**) of hot fresh pork under different transport temperature setpoint conditions. Different uppercase letters indicate significant differences among transportation distances at the same temperature (*p* < 0.05). Different lowercase letters indicate significant differences among transportation conditions at the same distance (*p* < 0.05). Abbreviations: GPa, Glycogen phosphorylase-a; PK, Pyruvate kinase.

Glycogen phosphorylase (GP), one of the key rate-controlling enzymes, exists in active (GPa) and inactive (GPb) forms. Glycogenolytic flux is primarily determined by GPa levels [46]. GP catalyzes glycogen degradation to glucose-1-phosphate, supplying an essential substrate for glycolytic metabolism [47]. In this study, GPa activity in hot fresh pork showed an initial increase followed by a decline during transportation (Figure 7A). This biphasic pattern reflects early post-slaughter conversion of GPb to GPa, succeeded by glycogen depletion, enzyme degradation, and energy exhaustion. The control group exhibited peak GPa activity at 50 km, which was higher (*p* < 0.05) than that in other groups, a phenomenon often associated with accelerated glycolytic process and rapid pH decline [48]. At 150 km, GPa activity of the 5 °C Setpoint group was higher than those of the 15 °C Setpoint and control groups (*p* < 0.05), potentially due to sustained catalysis requirement at higher glycogen levels under slowed glycolysis [49]. These results indicate that low-temperature transportation delayed the GPa activation, thereby suppressing glycolysis and regulating post-slaughter meat quality.

Pyruvate kinase (PK), another key glycolytic enzyme, regulates energy metabolism and meat quality by catalyzing the conversion of phosphoenolpyruvate (PEP) to pyruvate [50]. As shown in Figure 7B, PK activity decreased gradually during transportation. The control group exhibited higher terminal PK activity (*p* < 0.05) than the 5 °C and 10 °C Setpoint groups, corresponding to their elevated lactate accumulation. This observation aligns with reports that cooling suppresses metabolic enzyme activity, decelerating early post-mortem glycolysis and lactate accumulation [51]. The coordinated changes in the activities of the rate-limiting enzymes (GP and PK), together with the observed extent of glycogen depletion and lactate accumulation, provide direct evidence that glycolytic flux was modulated by transportation temperature. Consistent with these findings, lower temperature reduced PK activity, attenuated glycolysis and lactate accumulation, consequently leading to improved meat quality regulation.

Glycolysis profoundly influences the post-mortem quality of meat. As evidenced by our data, transportation temperature significantly modulated the activities of key glycolytic enzymes, glycogen phosphorylase (GP) and pyruvate kinase (PK) (Figure 7). The attenuated glycolytic flux observed under 5 °C and 10 °C Setpoint conditions, reflected in the suppressed activation of GP and decreased PK activity, led to a more gradual decline in post-mortem pH. The rate of pH decline is a critical determinant of myoglobin denaturation and the functional efficacy of the metmyoglobin reductase system [37]. Consequently, a slower pH fall helps preserve the activity of the metmyoglobin reductase, which contributes to stabilizing the redness (a* value) of the meat. In contrast, the control group exhibited accelerated glycolysis, which precipitated a rapid pH drop. This acidic milieu not only promotes myoglobin denaturation but also impairs the endogenous reducing capacity of the muscle, leading to rapid discoloration, which is a trend that is consistent with the observed decline in a* value. Furthermore, the variation in glycolytic rate and ultimate pH profoundly influences the physical structure of muscle. As demonstrated in the control group, the higher transportation temperature promoted glycolysis, resulting in a rapid pH decline. This can induce the denaturation of myofibrillar proteins such as myosin, which adversely affects water-holding capacity [52].

### 3.8. Microbial Community

#### 3.8.1. Bacterial Community Diversity

**Figure 8:**
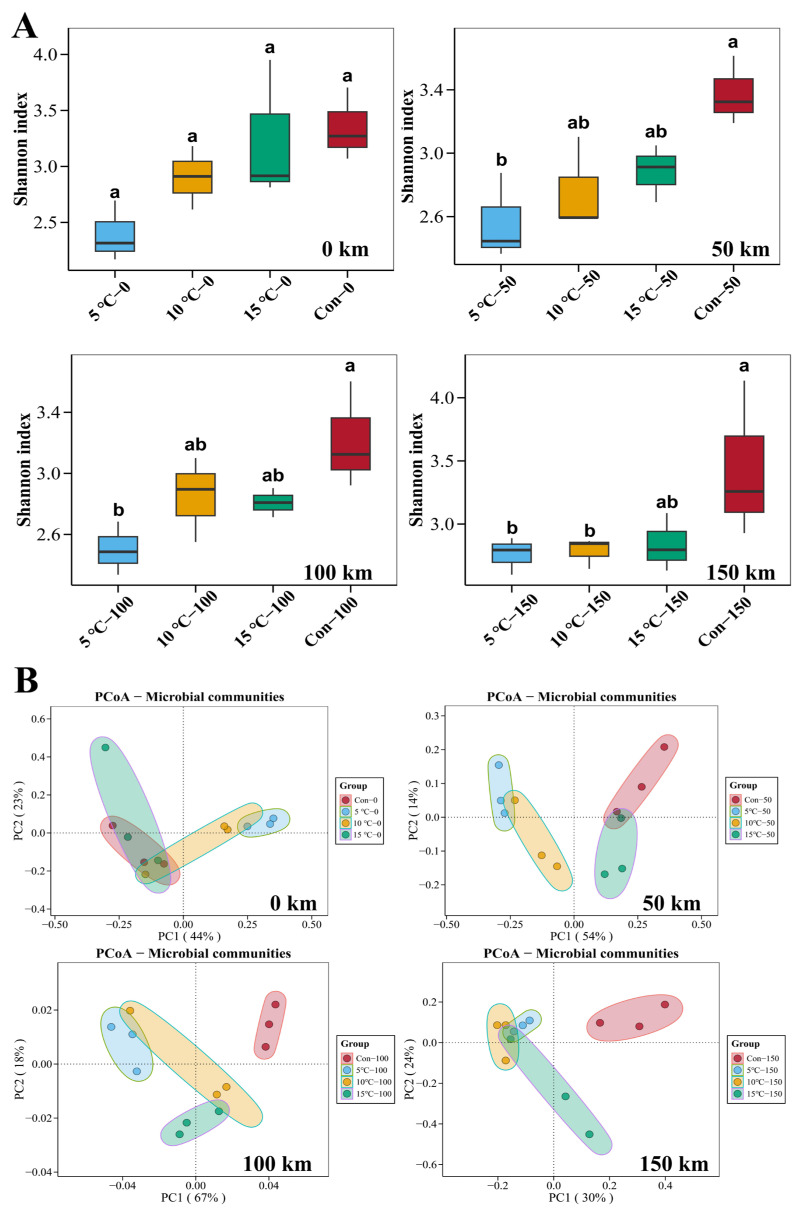
Effects of transportation temperature setpoint on bacterial community diversity in pork. Alpha diversity (Shannon index) (**A**); Beta diversity based on principal coordinates analysis (PCoA) (**B**). Note: 0 km (start of transportation at 5 °C, 10 °C, 15 °C Setpoint groups, and ambient temperature); 50 km (after 50 km transportation at 5 °C, 10 °C, 15 °C Setpoint groups, and ambient temperature); 100 km (after 100 km transportation at 5 °C, 10 °C, 15 °C Setpoint groups, and ambient temperature); 150 km (after 150 km transportation at 5 °C, 10 °C, 15 °C Setpoint groups, and ambient temperature). Different lowercase letters (a, b) indicate significant differences among transportation conditions at the same distance (*p* < 0.05).

This study utilized 16S rRNA high-throughput sequencing to analyze the bacterial diversity and community structure in the hot fresh pork during transportation. Alpha diversity, assessed using the Shannon index at the OTU level, increased with the number of species and the abundances in the sample. At 150 km, the Shannon index was higher (*p* < 0.05) in the control group than in the 5 °C and 10 °C Setpoint groups (Figure 8A), indicating that microbial diversity remained stable during cold chain transportation, whereas ambient conditions promoted a more complex bacterial community with increased abundance and diversity. Considering the higher microbial colony counts and accelerated TVB-N accumulation in the control group, these results demonstrate that transportation temperature significantly influences bacterial community structure, consequently elevating spoilage potential in ambient-transported pork.

Principal coordinates analysis (PCoA) was applied to assess dissimilarities in bacterial composition among samples. Microbial communities gradually clustered into distinct quadrants according to transportation temperatures (Figure 8B). Throughout transportation, communities showed dynamic changes. After 150 km, the control group clearly separated from the 5 °C, 10 °C, and 15 °C Setpoint groups, indicating distinct bacterial composition. Greater similarity was observed between the 5 °C and 10 °C Setpoint groups. These findings highlight the substantial effect of low temperature on the bacterial community composition of transported pork.

#### 3.8.2. Bacterial Community Composition

**Figure 9:**
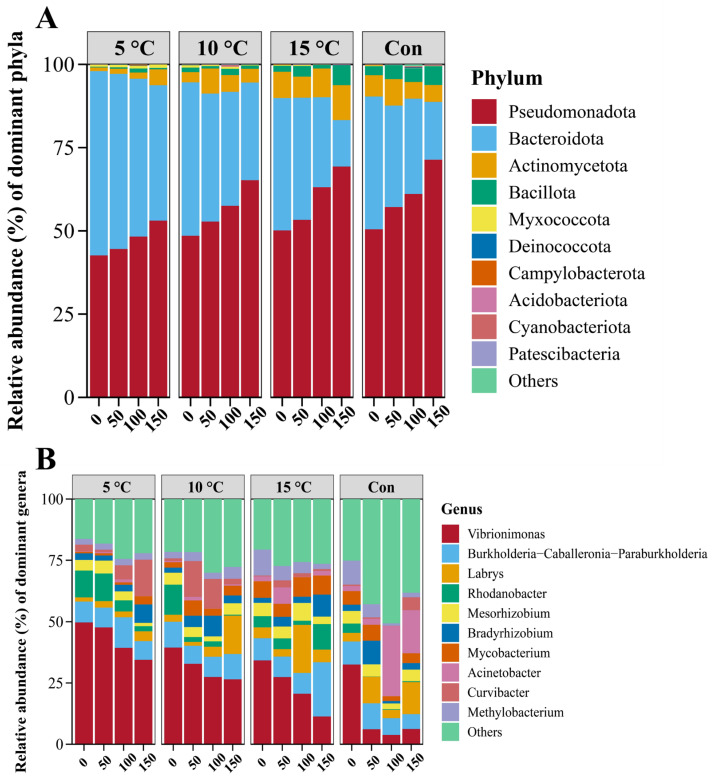
Changes in bacterial community composition and abundance under different transportation setpoint temperatures. Relative abundance at the phylum level (**A**); relative abundance at the genus level (**B**).

Figure 9A illustrates the dynamic changes in bacterial community abundance at the phylum level. At this level, five major phyla were identified: Pseudomonadota, Bacteroidota, Actinomycetota, Bacillota, and Myxococcota. Consistent with previous studies, Pseudomonadota and Bacillota were recognized as the predominant spoilage-related phyla in meat products [53]. Pseudomonadota was the most abundant phylum in the hot fresh pork and increased continuously during transportation, accounting for 53% in the 5 °C Setpoint group and 71% in the control group at the endpoint, suggesting a visibly faster increase under ambient conditions. Meanwhile, Bacillota represented 5.7–6.0% of the community in the 15 °C Setpoint and control groups at the endpoint, but only 0.4–1.0% in the 5 °C and 10 °C Setpoint groups, indicating that lower temperatures suppressed its growth. In contrast, Actinomycetota increased notably in the 5 °C and 10 °C Setpoint groups yet remained stable in the 15 °C and control groups. Bacteroidota was the second most dominant phylum in hot fresh pork and maintained high relative abundance across all groups. Minor phyla such as Myxococcota and Deinococcota were also detected in each group.

At the genus level, 465 genera were identified, with the top 10 shown in Figure 9B. *Vibrionimonas* was the dominant genus across all groups at 0 km, accounting for 49.7%, 39.4%, 34.2%, and 32.5% in the 5 °C Setpoint, 10 °C Setpoint, 15 °C Setpoint, and control groups, respectively. Other initial colonizers included *Burkholderia-Caballeronia-Paraburkholderia*, *Rhodanobacter*, *Mesorhizobium* and *Methylobacterium*, which are commonly associated with the slaughter and processing environments [54]. Throughout transportation, the abundance of *Vibrionimonas* decreased but remained predominant in the 5 °C and 10 °C Setpoint groups. At the endpoint, *Rhodanobacter* (10.4%) and *Mycobacterium* (7.7%) were more abundant in the 15 °C Setpoint group, whereas *Acinetobacter* (17.6%) and *Labrys* (13.1%) dominated in the control group. *Acinetobacter* is a known spoilage organism in pork, and its high abundance correlates with accelerated quality deterioration [55]. The control group also exhibited a higher collective abundance of low-abundance phyla, indicating that ambient-temperature transportation may increase community diversity, supporting the alpha diversity findings. It is worth noting that the dominant genera observed in this study differed from those reported in other pork storage models, such as the prevalence of *Lactobacillaceae* under stable chilled conditions. This divergence may be attributable to the fluctuating temperature regimes and the relatively short duration (3 h) of transportation in our study, which represent a distinct selective environment compared with long-term storage.

**Figure 10:**
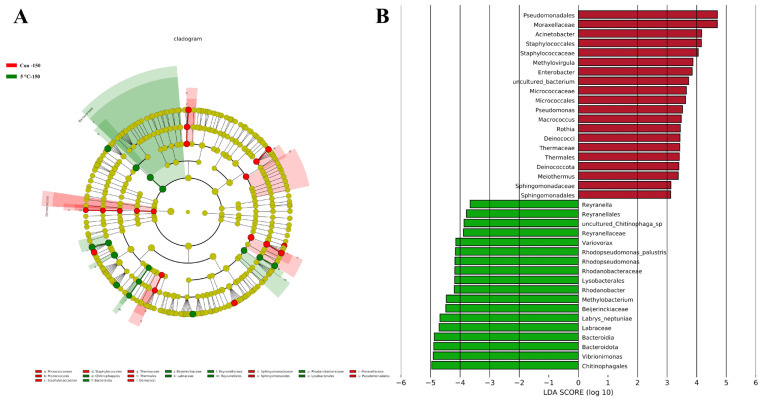
Microbial community analysis of pork meat using LEfSe. (**A**) Cladogram illustrating the phylogenetic distribution of microbial biomarkers. (**B**) Histogram of linear discriminant analysis (LDA) scores for taxa with LDA > 2 and Padj < 0.05. Note: Con-150 (ambient temperature transportation over 150 km); 5 °C-150 (transportation at the 5 °C setpoint over 150 km).

LEfSe analysis revealed a distinct microbiota composition in the 5 °C Setpoint group compared to the control group (Figure 10A). Pseudomonadales and Moraxellaceae were the most major biomarkers under ambient transportation conditions (Figure 10B). Pseudomonadales have been reported to produce volatile metabolites derived from amino acids catabolism, which contribute directly to meat spoilage [56]. Within the Moraxellaceae family, genera such as Acinetobacter and Psychrobacter have been implicated in meat spoilage via similar proteolytic and lipolytic pathways [57]. The presence of these pathogenic biomarkers may indicate that ambient temperature transportation could accelerate the proliferation of spoilage-related and potentially pathogenic microorganisms, thereby elevating food safety risk.

## 4. Conclusions

As transportation distance increased, hot fresh pork quality exhibited clear temperature-dependent changes. During the 150 km transportation, the 5 °C and 10 °C Setpoint groups effectively delayed glycogen degradation and ATP consumption. Concurrently, lactic acid accumulation was suppressed, leading to a slower pH decline. Water-holding capacity was also improved under low-temperature conditions, with reduced cooking loss and drip loss observed in the 5 °C and 10 °C Setpoint groups. Assessments of key freshness indicators showed that both microbial colony counts and TVB-N values were lower in the temperature-controlled groups. In terms of meat color, the 10 °C Setpoint group maintained higher a* values, whereas the 5 °C Setpoint group showed darker coloration due to excessive suppression of myoglobin oxygenation. Bacterial community analysis indicated that 10 °C Setpoint transportation inhibited the proliferation of spoilage-related taxa such as Pseudomonadota and Acinetobacter. In summary, transportation at 10 °C Setpoint achieved a favorable balance among color retention, protein degradation delay, and microbial control, suggesting a promising strategy for the long-distance distribution of hot fresh pork during summer. Future work should validate the temperature effects across diverse commercial conditions—including different genotypes, sexes, and age ranges—to confirm the generalizability of the temperature-controlled transportation strategy.

## Data Availability

The original contributions presented in the study are included in the article, further inquiries can be directed to the corresponding authors.
